# Supplementary material for: Solitary Fibrous Tumor/Hemangiopericytoma Metastasizes Extracranially, Associated with Altered Expression of WNT5A and MMP9
Source: Cancers (Basel). 2021 Mar 7;13(5):1142. doi: 10.3390/cancers13051142 (PMC7962064; doi:10.3390/cancers13051142)
Supplement: Supplementary file 1 [file cancers-13-01142-s001.pdf]

Supplementary Table S1. Baseline characteristics of the patients with primary SFT/HPC

		***ex*SFT/HPC (No. of patients = 32)**	***ic*SFT/HPC (No. of patients = 18)**
Age (yrs)	Median (range)	53 (23–79)	49 (22–78)
Sex	Male	13 (41%)	9 (50%)
	Female	19 (59%)	9 (50%)
Size (cm)	Median (range)	8.5 (1.7–21.0)	4.7 (1.5–7.0)
Histologic grade	Benign/grade 2	23 (72%)	9 (50%)
	Malignant/grade 3	9 (28%)	9 (50%)
Post-operative treatment	Chemotherapy	1 (3%)	1 (6%)
	Radiation	3 (9%)	10 (56%)
Recurrence	Local	3 (9%)	5 (28%)
	Distant	2 (6%)	3 (17%)

*ic*SFT/HPC, intracranial solitary fibrous tumor/hemangiopericytoma; *ex*SFT/HPC, extracranial solitary fibrous tumor/hemangiopericytoma.

Supplementary Table S2. Clinical information on intracranial SFT/HPC cases with extracranial metastsis

	Case 1	Case 2	Case 3	Case 4	Case 5	Case 6
Age(yrs)/ Sex	34/F	55/F	39/M	36/F	57/F	29/F
Intracranial location	Right tentorium	Sphenoid ridge	Right tentorium	Right cerebellum	Occipital lobe	Temporal lobe
Metastatic organ	Liver	C7 vertebrae	T9 vertebrae	Lung	Lung	Pelvic soft tissue
Initial tumor grade	Grade 2	NA	Grade 3	Grade 2	Grade 3	Grade 3
Duration to metastasis (months)	216	116	45	65	32	84

Supplementary Figure S1.

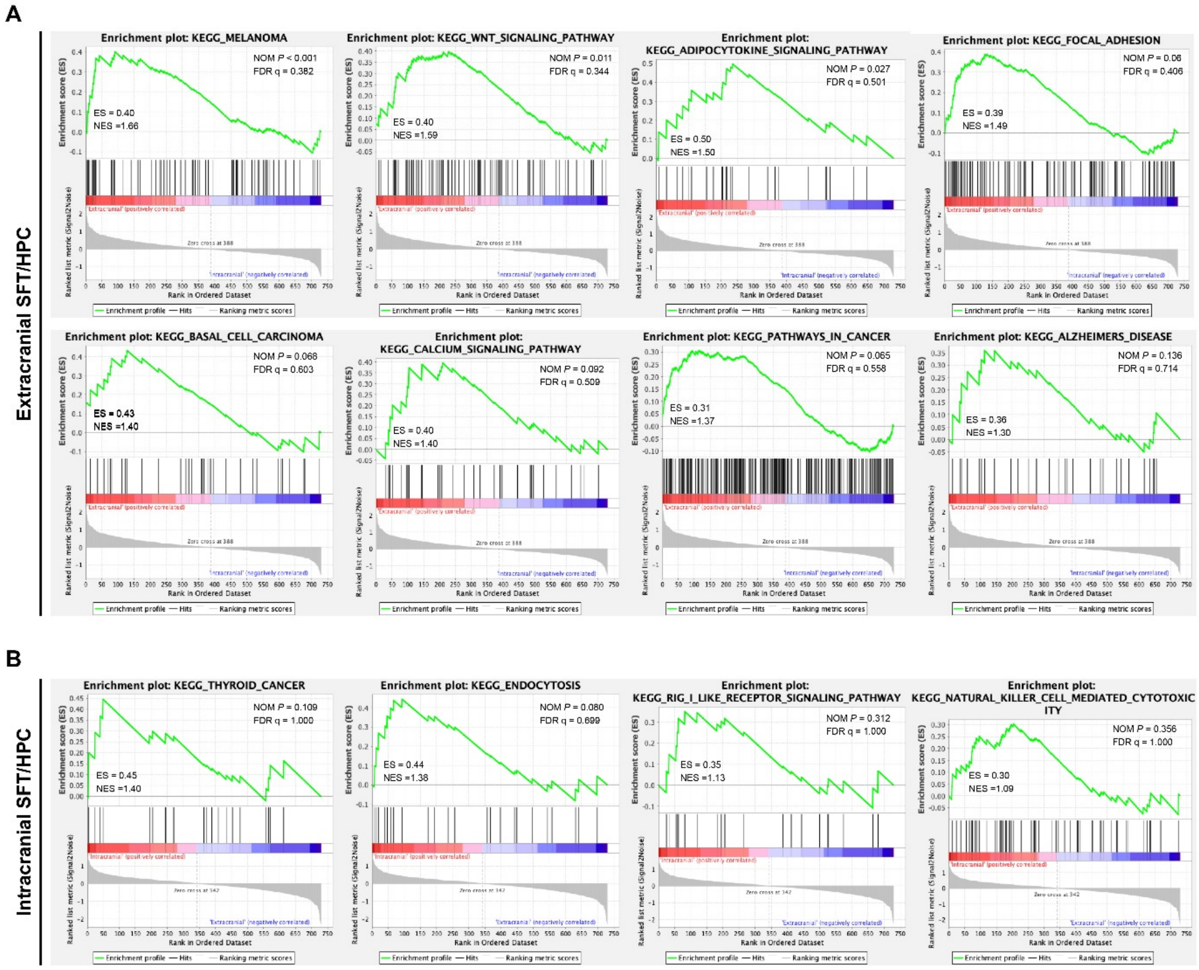

Supplementary Figure S1. Gene set enrichment analysis (GSEA) enrichment plot (score curves) using Nanostring data comparing *ic*SFT/HPC and *ex*SFT/HPC. (A) KEGG gene sets enriched in *ex*SFT/HPC. (B) KEGG gene sets enriched in *ic*SFT/HPC.

Supplementary Figure S2.

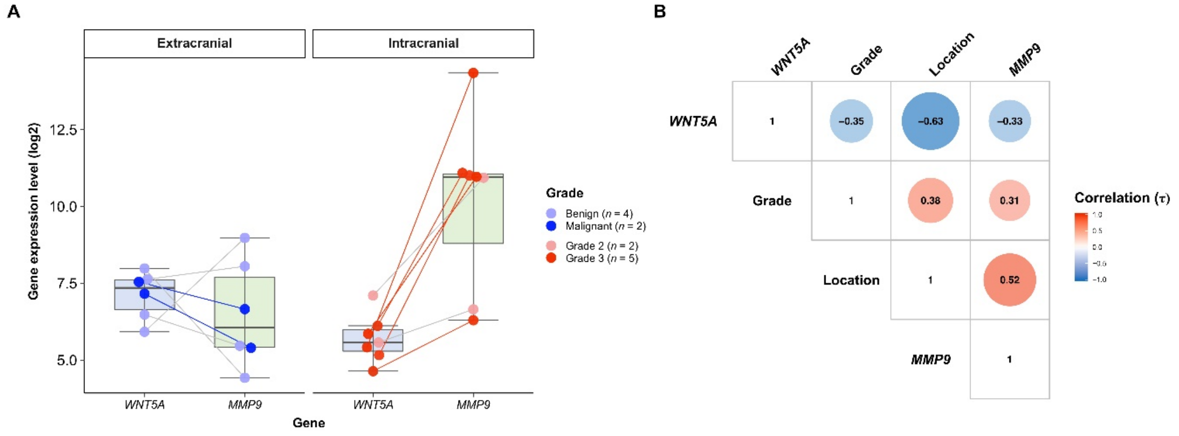

Supplementary Figure S2. Differential expression of *WNT5A* and *MMP9* according to tumor location and grade. (A) Paired boxplots of *WNT5A* and *MMP9* showed that the difference in the expression of *WNT5A* and *MMP9* was greater in *ic*SFT/HPC compared to *ex*SFT/HPC. (B) Correlogram showing that expression of *WNT5A* was positively correlated with an extracranial location and that expression of *MMP9* with an intracranial location. The intracranial location was coded as a positive value, and the extracranial location as a negative value.

Supplementary Figure S3.

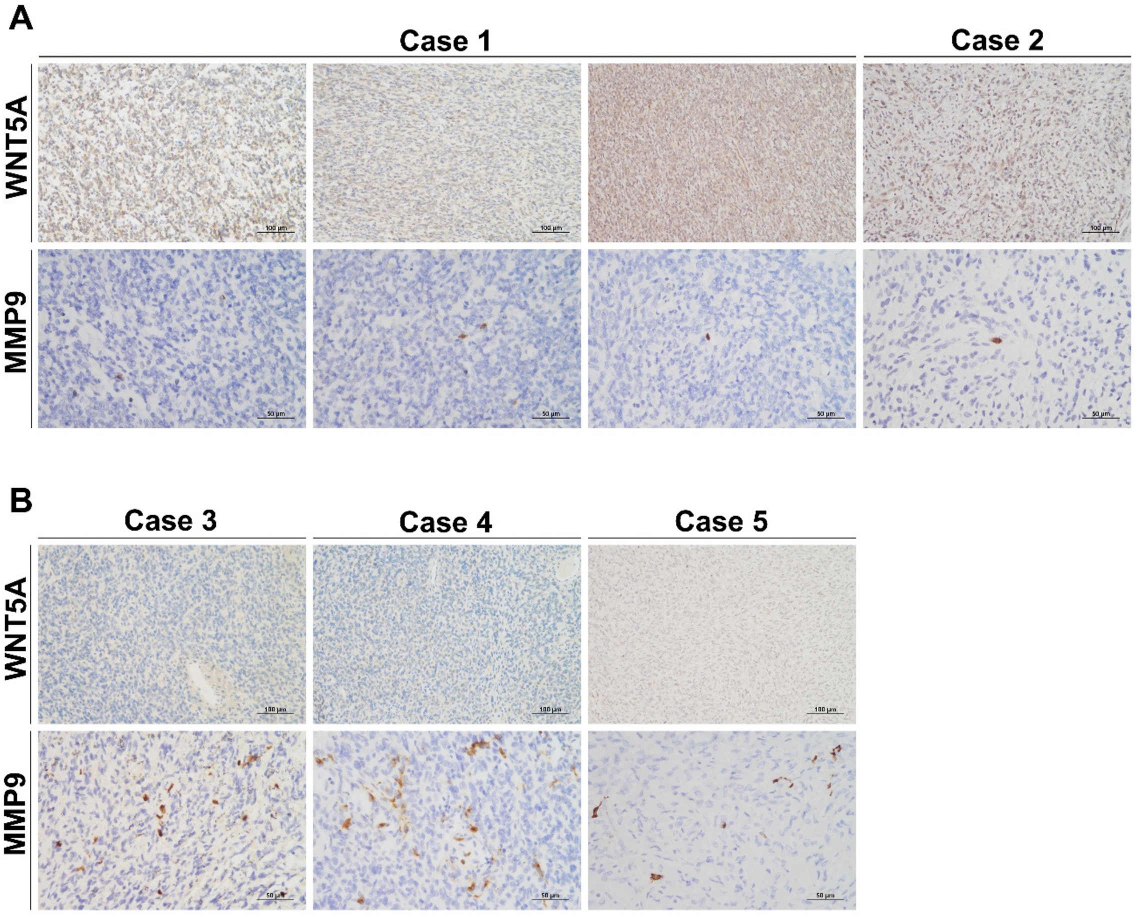

Supplementary Figure S3. Immunohistochemistry of WNT5A and MMP9 in cases of *ex*SFT/HPC and *ic*SFT/HPC exhibiting local recurrence. (A) Two cases of *ex*SFT/HPC (cases 1 and 2) with local recurrence maintained high expression of WNT5A and low expression of MMP9. Case 1 of *ex*SFT/HPC showed multiple recurrences, and tumor samples were obtained three times after initial resection. (B) Three cases of *ic*SFT/HPC (cases 3, 4, and 5) with local recurrence revealed low expression of WNT5A and high expression of MMP9.
